# Benign Proliferative Nodules in a Giant Congenital Melanocytic Nevus: Diagnostic Challenge and Management

**DOI:** 10.1155/crdm/9996929

**Published:** 2026-10-18

**Authors:** Fenohasina Rakotonandrasana, Holy Tiana Andrianjafitrimo, Onivola Raharolahy, Andrianandrianina Mbolatiana Kiady Armando Rakotomanana, Georges Cheny Fenohasina, Voahanginirina Nathalie Ralimalala, Malalaniaina Andrianarison, Fandresena Arilala Sendrasoa, Irina Mamisoa Ranaivo, Fahafahantsoa Rapelanoro Rabenja, Lala Soavina Ramarozatovo

**Affiliations:** ^1^ Department of Dermatology Joseph Raseta Befelatanana Hospital University of Antananarivo Antananarivo Madagascar univ-antananarivo.mg; ^2^ Anatomic and Pathological Cytology Department Joseph Ravoahangy Andrianavalona Hospital University of Antananarivo Antananarivo Madagascar univ-antananarivo.mg; ^3^ Department of Dermatology Place Kabary University of Antsiranana Antsiranana Madagascar

**Keywords:** congenital melanocytic nevus, infant, nevus, pigmented, skin neoplasms

## Abstract

Congenital melanocytic nevi are cutaneous malformations present at birth, resulting from a benign proliferation of melanocytes. Although generally benign, they can have significant psychological impacts and carry a non‐negligible risk of progression to malignant melanoma. We report a case of a giant congenital melanocytic nevus associated with a perianal mass in an infant, two and half‐month‐old infant, with no notable medical history, born at term. He was referred for a pigmented perianal swelling located on a large hyperpigmented plaque covering the lower back, buttocks, perineum, external genitalia, and the upper third of the thighs, consistent with a giant nevus. Multiple pigmented maculopapular satellite lesions were present, extending from the scalp to the soles of the feet. All lesions had been present since birth. The general examination was unremarkable. Doppler ultrasound revealed a well‐defined, homogeneous, hypoechoic subcutaneous lesion with low vascularity, and skin biopsy confirmed a benign proliferative nodule. The proposed management included surgical excision, neurological and growth development follow‐up, and psychosocial support for the infant and the parents. Six months after surgery, follow‐up showed no evidence of recurrence, with the infant demonstrating normal neurological function and appropriate growth and developmental progress. This case highlights the importance of an accurate clinical and histological diagnosis and a multidisciplinary approach to management. It contributes to the limited local data from Madagascar and may contribute to the understanding of potential complications and their management.

## 1. Introduction

Congenital melanocytic nevi are cutaneous malformations present at birth, resulting from a benign proliferation of melanocytes. They are most commonly located on the trunk and are frequently associated with hypertrichosis and the presence of satellite nevi, particularly in giant congenital melanocytic nevi, where multiple satellite lesions represent a characteristic clinical feature [1–3]. They occur in approximately 1%‐2% of newborns, while the incidence of giant congenital melanocytic nevi is estimated at 1 in 20,000 births [2, 4]. Although benign in most cases, congenital melanocytic nevi can have significant psychological and behavioral impacts and carry a noteworthy risk of progression. The risk of malignant transformation into melanoma is substantially higher in giant congenital melanocytic nevi than in small or medium sized lesions, with an estimated lifetime risk of approximately 3%–8%, while 5%–8% are associated with neurocutaneous melanocytosis [5–7].

Benign proliferative nodules may also arise within congenital melanocytic nevi, particularly in giant lesions, and may present with rapid growth and clinical or histopathological features that overlap with those of melanoma, posing a diagnostic challenge [8, 9]. In Madagascar, data on congenital melanocytic nevi remain extremely limited, which, together with the potential diagnostic difficulty posed by proliferative nodules, prompted us to report this case.

## 2. Case Report

Two and half‐month‐old male infant, the third child in a family of three, with no similar family history and no notable medical conditions. He was born at term by vaginal delivery after 38 weeks of gestation, from a nonconsanguineous pregnancy, with good neonatal adaptation. He was referred for a pigmented perianal swelling present since birth. Dermatological examination revealed a well‐defined pigmented tumoral lesion of soft to firm consistency, located on the right perianal region. The lesion extended over a large, well‐demarcated hyperpigmented plaque involving the lower back, buttocks, perineum, external genitalia, and the upper third of the thighs, measuring more than 20 cm, consistent with a giant nevus (Figures 1 and 2). It was associated with multiple pigmented maculopapular satellite lesions of variable size (≤ 4 cm), diffusely distributed from the scalp to the soles (> 25 nevi), all of which had also been present since birth. The remainder of the examination revealed a calm, afebrile infant, with normal defecation, no palpable lymphadenopathy, and normal neurological, cardiac, and pulmonary findings. Doppler ultrasonography of the mass revealed a 15 × 9 mm homogeneous hypoechoic subcutaneous lesion, regular and well circumscribed, with low vascularization and no adjacent inflammatory infiltration.

**FIGURE 1:**
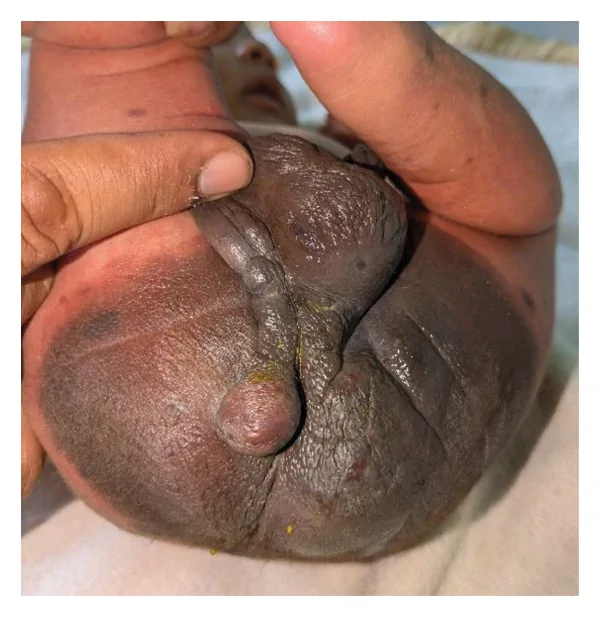
Well‐circumscribed 2 × 1 cm right perianal tumoral lesion overlying a giant congenital melanocytic nevus. *Source*: department of dermatology, Joseph Raseta Befelatanana, Antananarivo, Madagascar.

**FIGURE 2:**
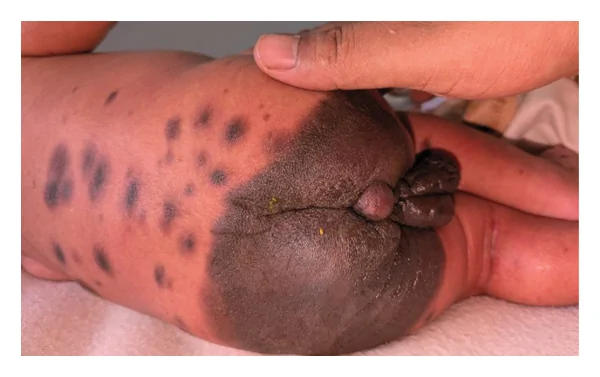
Tumoral lesion on a giant congenital melanocytic nevus with multiple satellite nevi on the back. *Source*: department of dermatology, Joseph Raseta Befelatanana, Antananarivo, Madagascar.

A skin biopsy of the perianal tumoral lesion showed acanthosis with mild papillomatosis. Melanocytes were present in the epidermis and superficial dermis, without cytological atypia. The overall architecture was preserved. These findings are consistent with a dermal nevus (Figure 3), supporting the diagnosis of a benign proliferative nodule. Therapeutic management included a proposed surgical excision of the tumor, along with psychological support and monitoring of neurological status and growth and developmental progress. At 6 months postsurgery, follow‐up evaluation showed no signs of recurrence, with the patient maintaining normal neurological function and satisfactory growth and developmental milestones.

**FIGURE 3:**
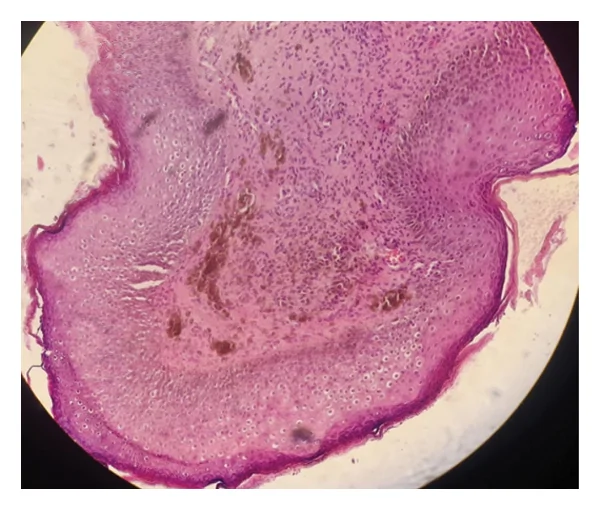
Histological section of a perianal skin biopsy (hematoxylin and eosin stain, × 20 magnification) showing epidermal acanthosis with mild papillomatosis and a benign melanocytic proliferation involving the epidermis and superficial dermis, without histological evidence of malignancy. *Source*: anatomic and pathological cytology department, Joseph Ravoahangy Andrianavalona Hospital, Antananarivo, Madagascar.

## 3. Discussion

Congenital melanocytic nevi are rare conditions. Somatic mutations involving BRAF, NRAS, and RAF play a central role in their pathogenesis. These genetic alterations activate the MAPK and PI3K‐AKT signaling pathways, disrupting the normal development of melanoblasts during embryogenesis and leading to the formation of nevi [10, 11]. Their prevalence is estimated at 1%‐2% of births, with a slight female predominance. Severe forms, such as large and giant nevi, remain rare [2, 4]. Large congenital nevi can present with proliferative nodules, which may pose a significant histopathological diagnostic challenge because they can mimic congenital melanoma, although this complication remains rare. Benign proliferative nodules are generally characterized by increased melanocytic cellularity while preserving relatively uniform cytological features, low mitotic activity, and the absence of marked nuclear atypia, frequent or atypical mitotic figures, and necrosis and infiltrative growth, making careful clinicopathological correlation essential for an accurate diagnosis. Melanoma can occur at any age, including in young children, and its deep development may complicate diagnosis [7, 12]. Giant congenital melanocytic nevi are associated with an increased risk of neurocutaneous melanocytosis, and brain and spinal MRI should be considered during follow‐up, particularly in patients with multiple satellite nevi or neurological symptoms, to facilitate early detection and appropriate management [13, 14]. A Brazilian study by Viana et al., including 57 patients with giant congenital melanocytic nevi, predominantly children (median age at study entry: 2.6 years; 62.4% were younger than 5 years), reported a melanoma incidence of 3.5% during follow‐up, highlighting the importance of careful long‐term surveillance in this high‐risk population [15].

Proliferative nodules represent a benign hyperplasia of melanocytes and are often associated with focal thickening of the skin or a localized tumoral lesion. Histologically, they typically exhibit preserved architecture, without nuclear atypia or abnormal mitoses [2, 4, 7].

In contrast, congenital melanoma is characterized by significant cytological atypia, frequent or atypical mitotic figures, areas of necrosis, and destructive or infiltrative growth [7, 14]. Management relies on an accurate diagnosis combining clinical examination and histology. Therapeutic options include surgical excision of symptomatic nodules, regular dermatological follow‐up, and supportive measures for the skin and quality of life, such as pruritus management and prevention of skin trauma. Psychosocial support for patients and their families is essential, given the esthetic and psychological impact of these lesions [2, 5–7]. Finally, recent publications suggest that targeted therapies, such as locally applied MEK or c‐KIT inhibitors, could represent a future option to reduce melanocytic proliferation and prevent complications in extensive congenital nevi although these approaches remain experimental and limited to preclinical models [10, 11, 16]. In Madagascar, limitations in diagnostic and therapeutic resources make clinical evaluation essential. Interruptions in follow‐up further complicate patient management and long‐term surveillance although psychosocial support for patients and their families remains feasible. Patient education should be promoted, including self‐monitoring, photoprotection, and coping with social perception. Finally, a multidisciplinary approach is essential, involving dermatologists, surgeons, psychologists, neurologists, and pediatricians, to ensure comprehensive patient care.

Giant congenital nevi present significant diagnostic and therapeutic challenges due to their complex natural history, including the risk of malignant transformation and associated neurological involvement. Proliferative nodules arising in giant congenital melanocytic nevi also present significant diagnostic challenges due to their complex clinical and histopathological features, particularly their potential to mimic melanoma. This case contributes to the limited local data on congenital melanocytic nevi and associated lesions, highlights the challenges encountered in resource‐limited settings, and underscores the importance of a comprehensive, multidisciplinary, and patient‐centered approach, including education of patients and their families.

**Nomenclature**
MRI: Magnetic resonance imaging

## Author Contributions

Conception and design: Fenohasina Rakotonandrasana, Georges Cheny Fenohasina, Andrianandrianina Mbolatiana Kiady Armando Rakotomanana, and Voahanginirina Nathalie Ralimalala.

Review and editing: Fenohasina Rakotonandrasana, Holy Tiana Andrianjafitrimo, Onivola Raharolahy, Malalaniaina Andrianarison, and Fandresena Arilala Sendrasoa.

Final approval of the version to be published: Irina Mamisoa Ranaivo, Lala Soavina Ramarozatovo, and Fahafahantsoa Rapelanoro Rabenja.

## Funding

The authors have nothing to report.

## Consent

No written consent has been obtained from the patients as there are no patient identifiable data included in this case report.

## Conflicts of Interest

The authors declare no conflicts of interest.

## Data Availability

The data that support the findings of this study are available on request from the corresponding author.
